# Genome-wide association analysis of pulse wave velocity traits provide new insights into the causal relationship between arterial stiffness and blood pressure

**DOI:** 10.1371/journal.pone.0237237

**Published:** 2020-08-13

**Authors:** Michael Rode, Andrej Teren, Kerstin Wirkner, Katrin Horn, Holger Kirsten, Markus Loeffler, Markus Scholz, Janne Pott

**Affiliations:** 1 Institute for Medical Informatics, Statistics and Epidemiology, University of Leipzig, Leipzig, Germany; 2 LIFE Research Center for Civilization Diseases, University of Leipzig, Leipzig, Germany; 3 Heart Center Leipzig, Leipzig, Germany; 4 Leipzig University Medical Center, IFB Adiposity Diseases, Leipzig, Germany; Medizinische Universitat Graz, AUSTRIA

## Abstract

**Background:**

The pathophysiology of arterial stiffness is not completely understood. Pulse wave velocity (PWV) is an established marker for arterial stiffness. We compare genetics of three PWV modes, namely carotid-femoral PWV (cfPWV), brachial-ankle (baPWV) and brachial-femoral (bfPWV), reflecting different vascular segments to analyse association with genetic variants, heritability and genetic correlation with other biological traits. Furthermore we searched for shared genetic architecture concerning PWV, blood pressure (BP) and coronary artery disease (CAD) and examined the causal relationship between PWV and BP.

**Methods and results:**

We performed a genome-wide association study (GWAS) for cfPWV, baPWV and bfPWV in LIFE-Adult (N = 3,643–6,734). We analysed the overlap of detected genetic loci with those of BP and CAD and performed genetic correlation analyses. By bidirectional Mendelian Randomization, we assessed the causal relationships between PWV and BP. For cfPWV we identified a new locus with genome-wide significance near *SLC4A7* on cytoband 3p24.1 (lead SNP rs939834: p = 2.05x10^-8^). We replicated a known PWV locus on cytoband 14q32.2 near *RP11-61O1*.*1* (lead SNPs: rs17773233, p = 1.38x10^-4^; rs1381289, p = 1.91x10^-4^) For baPWV we estimated a heritability of 28% and significant genetic correlation with hypertension (rg = 0.27, p = 6.65x10^-8^). We showed a positive causal effect of systolic blood pressure on PWV modes (cfPWV: p = 1.51x10^-4^; bfPWV: p = 1.45x10^-3^; baPWV: p = 6.82x10^-15^).

**Conclusions:**

We identified a new locus for arterial stiffness and successfully replicated an earlier proposed locus. PWV shares common genetic architecture with BP and CAD. BP causally affects PWV. Larger studies are required to further unravel the genetic determinants and effects of PWV.

## Introduction

Cardiovascular disease (CAD) was shown to be a heritable disease and genome-wide association analyses so far discovered 169 loci [1–5]. Epidemiologic studies suggest that arterial stiffness is an independent predictor for cardiovascular risk and can be considered as an intermediate phenotype [6, 7]. Pulse wave velocity (PWV) has been proposed as a reliable method to measure arterial stiffness [8, 9] and reference curves for carotid-femoral PWV (cfPWV), brachial-ankle PWV (baPWV) and brachial-femoral PWV (bfPWV) were established accordingly [10]. Heritability of PWV was shown to be moderate in between 0.11 and 0.40 for cfPWV [11, 12].

Beside genetic pre-disposition, biological aging causes arterial stiffening due to structural changes in the medial layer of the arterial walls [13]. While for many years hypertension was considered as causal for arterial stiffness, during the last decade several studies showed an inconclusive picture suggesting a reverse causality [14].

As so far no GWAS measured all three PWV modes in parallel on individuals of a large population-based study, we gain new insights on how the underlying genetic factors affect different segments of the arterial tree. Furthermore we analyse the causal relationship between arterial stiffness and blood pressure as well as the common genetic effects of PWV, blood pressure and CAD.

## Material and methods

### Cohort description

LIFE-Adult is a randomly sampled population based study of 10,000 participants from Leipzig, a city in the east of Germany [15]. Most of the participants are aged between 40 and 79, with a small sub group of 400 participants being between 18 and 39. The study population is of central European descent and the main study goal is to investigate prevalence, genetic predisposition and the role of lifestyle-related factors (such as smoking habits, alcohol consumption, dietary patterns and physical activity) on major civilization diseases including subclinical signs. Initial data collection was performed between 2011 and 2014. LIFE-Adult meets the ethical standards of the Declaration of Helsinki. It has been approved by the Ethics Committee of the Medical Faculty of the University Leipzig, Germany (LIFE-Adult: Reg. No 263-2009-14122009). Written informed consent including agreement with genetic analyses was obtained from all participants.

### Pulse wave assessment

Pulse wave analysis was performed using the oscillometry-based Vicorder device (Vicorder, Skidmore medical, Bristol, UK) as previously described [8]. Validity and intra- and inter-observer reliability of the Vicorder device were shown to be good [9]. As recommended in Baier et al. [10], we used the first measurement available for baPWV and cfPWV while for bfPWV, we averaged values for first and second measurement, since variance of bfPWV was significantly higher than it was for baPWV and cfPWV. Measurements were discarded if the difference between first and second measurement was >5m/s (baPWV), >10m/s (cfPWV) and >25m/s (bfPWV), respectively. PWV modes were only moderately correlated. Considering systolic blood pressure (SBP) as confounder, partial correlations are 0.51 for bfPWV/cfPWV, 0.49 for baPWV/bfPWV and 0.37 for baPWV/cfPWV.

### Genotyping

For the genotyping of 7,838 randomly selected samples, we used the genome-wide SNP array Affymetrix Axiom CEU1, and for calling, Affymetrix Power Tools (APT) Version 1.20.6. We executed the calling according to the Affymetrix Axiom Genotyping Solution Data Analysis Guide and filtered 116 samples failing the dish QC criteria (dish QC ≥ 0.82, technology specific-signal-to-noise-ratio) and sample call rate criteria (SCR ≥ 97%) in the initial calling round [16]. Sex-mismatches were filtered and cryptic relatedness was assessed according to Wang [17]. Ten highly related samples (coefficient of relatedness > 0.99) were filtered. For X-chromosomal analysis, we filtered irregularities of X-Y intensity plots. Genetic principal components (“Swap-One-In”-PCA) were calculated using PLINK 1.9 and we removed ethnical outliers (>6 SD in any of first 10 principal components). For SNP quality control, the following filters were applied: SNP call rate ≥ 97%; p-value of exact test for Hardy-Weinberg equilibrium ≥10^−6^; p-value of plate association ≥10^−7^ (i.e. test for dependency of allele frequency on plate); monomorphic SNPs and criteria of cluster plot quality as suggested by Affymetrix. For X-chromosomal SNPs, call rate threshold was increased to 98% and HWE test performed in women was required to achieve p≥10^−4^. After QC of the initial 568,519 autosomal and 16,384 SNPs on chromosome X, a set of 7,669 LIFE-Adult subjects and 532,676 SNPs was available for autosomal analyses. For gonosomal analyses, 7,660 samples with 13,476 SNPs were used.

Genotype imputation was performed using 1000Genomes Project Phase 3, Version 5 [18] as reference and IMPUTE2 [19] as software.

After imputation, we excluded SNPs with minor allele frequency (MAF) <0.01 or IMPUTE info score <0.5 resulting in a total of 11,342,744 SNPs (thereof 358,330 SNPs gonosomal) used for the present analysis. Our sample sizes were n(baPWV) = 6,734, n(cfPWV) = 6,430 and n(bfPWV) = 3,643 [S1 Fig in S1 File].

### Statistical analysis

#### Genome-wide association analysis

Pulse wave parameters and blood pressures were log-transformed for all analyses to approximately conform to normality [S1 Table in S2 File]. We performed genome-wide association analyses for each phenotype by linear regression models assuming an additive mode of inheritance with adjustments for sex, age and SBP [S2 Table in S2 File]. Adjustment for SBP was included to avoid identifying blood pressure related SNPs based on the observed correlation between SBP and PWV (all Pearson’s R with p-values < 2.2x10^-16^: cfPWV: R = 0.24, bfPWV: R = 0.19; baPWV: R = 0.49) [20]. SBP was log transformed to achieve normal distribution. We tested the log transformed PWV modes for correlation with the first ten genetic principal components (PC). While there was no significant correlation with cfPWV, there was significant correlation with baPWV (PC8: p = 7.3x10^-4^, PC9: p = 7.0x10^-3^ and PC10: p = 8.3x10^-4^) and bfPWV (PC7: p = 2.6x10^-2^, PC9: p = 2.5x10^-4^ and PC10: 8.9x10^-2^). As in the multivariate model neither PC9 nor PC10 were significant for baPWV (PC9: p = 4.4x10^-1^ and PC10: 7.3x10^-1^) or bfPWV (PC9: p = 4.0x10^-1^ and PC10: 9.1x10^-1^), we decided to disregard the PC for all three PWV mode to apply a uniform model to all three modes. We tested the association of antihypertensive and lipid lowering medication on PWVs. They are associated with cfPWV and bfPWV, but do not explain much of the traits variance (r^2^<1%). Therefore, we did not adjust our genetic model for medication. We also considered log-transformed BMI as an additional covariate. Although significant, it explained only a small proportion of phenotype variance. Therefore we did not include it as additional adjustment. Analyses were executed with SNPTEST [21] using expected genotype counts. The threshold for genome-wide significance was set to p<5x10^-8^. Associations with p<1x10^-6^ were considered as suggestive and presented as list of top SNPs. In order to determine independent hits, we calculated linkage disequilibrium (LD) between markers in the LIFE-Adult data. Variants, which are in LD of r^2^≥0.5 with a SNP of higher significance, were considered tagged by that SNP (priority pruning). As locus we defined SNPs in a region of ±500 kb around the target SNP. Our approach had more than 80% power to detect an association of a SNP with more than 0.62% variance of our phenotype for cfPWV, which is quite well in the range of previously reported associations of comparable phenotypes in a similarly sized study [22].

A comprehensive annotation was applied to all SNPs of our top list using the following bioinformatics resources: Physically nearby genes were looked up from Ensemble [23] [13.09.2018] (up to four genes within ±250 kb). For associated variants, we assigned other GWAS hits in LD (r^2^>0.3) retrieved from the GWAS catalogue [24] [13.09.2018] and expression quantitative trait loci (eQTL) of several tissues [25–33] as well as our own data (LIFE eQTL data, see supplement for further explanation). Combined Annotation-Dependent Depletion (CADD) was used to measure variant deleteriousness [34].

#### Replication of known PWV SNPs

We looked up known and suggestive genetic loci for PWV and compared the reported effect sizes with our data [22, 35–37]. While the look-up of associated loci is feasible, the comparison of beta estimates is difficult since the other groups did not only use different PWV modes but also different transformations of their data. As our study is the first that performed a GWAS for three PWV modes in parallel, we provide new insights into the comparability of PWV assessments.

As of March 2019 four GWAS studies on PWV had been published [22, 35–37]. From these studies we selected SNPs for replication that had passed at least suggestive significance level (p<1.00x10^-6^) in the screening cohort [Table 1]. Mitchell et al. [35] and Tarasov et al. [22] identified ten (rs17773233, rs1381289, rs7152623, rs2225442, rs1461587, rs987514, rs10782490, rs1381273, rs8015529, rs10764094) respectively two SNPs (rs1194820, rs3742207) exceeding suggestive significance level, both using cfPWV on European populations. Park et al. [36], using baPWV on an Asian population, identified two SNPs (rs7271920, rs10125157) exceeding suggestive significance level. While they rejected the SNPs as they were not able to show nominal significance in their replication cohort, we included them for replication. Levy et al. (using cfPWV on a North American population) did not identify SNPs that passed at least suggestive significance level [37]. Therefore we identified in total 14 SNPs for replication analysis in our cohort. These SNPs are distributed over six loci. All of these SNPs were available in our data set. SNPs were considered as replicated in our study if at least Bonferroni corrected nominal significance (p<0.05/3) for any of the PWVs modes is achieved. For Mitchell et al. [35] the effect direction was expected to be opposite to ours as he considered 1000/cfPWV as primary phenotype. Authors used different regression models for their analysis. While Mitchel used a sex specific regression model with 1000/cfPWV as residual adjusted for age, age2, height and weight, Tarasov used inverse normal transformed cfPWV residuals adjusted for age, age^2^ and sex. Park used log transformed baPWV data, which adjusted for age, gender, systolic blood pressure and diastolic blood pressure. All models used different transformation for the residuals and did not deliver relevant higher r^2^ for our data [S2 Table in S2 File].

**Table 1:** Results of replication analysis: We considered SNPs for which associations with PWV modes were reported in the literature with at least suggestive significance level.

Known PWV SNPs and their characteristics in the original publication								Characteristics in LIFE Adult					
Cytoband	SNP	Source	EA	EAF	Beta	p-value	Phenotype	Best Phenotype	Tag SNP	EA	EAF	Beta	p-value
1p36.22	rs1194820	[22]	G	0.77	-0.11	3.48x10^-7^	cfPWV	bfPWV	no	G	0.73	0.019	1.69x10^-2^
9q33.1	rs10125157	[36]	n/a	n/a	-0.06	8.25x10^-7^	baPWV	cfPWV	yes	C	0.05	0.004	6.83x10^-1^
10p12.31	rs10764094	[35]	C	0.47	-0.06	2.40x10^-7^	cfPWV	bfPWV	no	C	0.49	-0.007	3.40x10^-1^
13q34	rs3742207	[22]	T	0.56	-0.09	5.16x10^-8^	cfPWV	baPWV	no	G	0.33	0.003	1.11x10^-1^
14q32.2	rs10782490	[35]	C	0.47	-0.07	2.70x10^-9^	cfPWV	baPWV	no	C	0.49	0.006	4.59x10^-3^
14q32.2	rs1381273	[35]	T	0.47	-0.06	1.90x10^-7^	cfPWV	baPWV	yes	T	0.46	0.004	2.73x10^-2^
**14q32.2**	**rs1381289**	**[35]**	**T**	**0.44**	**-0.07**	**5.60x10**^**-11**^	**cfPWV**	**baPWV**	**yes**	**T**	**0.43**	**0.008**	**1.91x10**^**-4**^
14q32.2	rs1461587	[35]	G	0.26	-0.07	1.50x10^-7^	cfPWV	baPWV	no	G	0.22	0.008	1.15x10^-3^
**14q32.2**	**rs17773233**	**[35]**	**T**	**0.23**	**-0.07**	**2.10x10**^**-8**^	**cfPWV**	**baPWV**	**yes**	**T**	**0.21**	**0.009**	**1.38x10**^**-4**^
14q32.2	rs2225442	[35]	C	0.32	-0.07	1.20x10^-8^	cfPWV	baPWV	no	C	0.40	0.007	7.98x10^-4^
14q32.2	rs7152623	[35]	A	0.38	-0.08	3.10x10^-15^	cfPWV	baPWV	no	A	0.38	0.007	2.47x10^-4^
14q32.2	rs8015529	[35]	G	0.36	-0.07	2.50x10^-7^	cfPWV	baPWV	no	G	0.34	0.004	3.56x10^-2^
14q32.2	rs987514	[35]	T	0.44	-0.07	4.50x10^-10^	cfPWV	baPWV	no	T	0.41	0.006	1.54x10^-3^
20p12.3	rs7271920	[36]	n/a	n/a	-0.17	7.20x10^-9^	baPWV	cfPWV	yes	T	0.12	-0.012	6.55x10^-2^

SNPs highlighted with grey back ground were not successfully replicated in our GWAS as they did not pass the Bonferroni corrected nominal significance level (p<1.67x10^*-2*^). SNPs in 14q32.2 are in LD with two independent lead SNPs (rs1381289 and rs17773233). Effect directions are opposite to Mitchell as he considered1000/cfPWV as phenotype. Summary statistics of all SNPs and phenotypes can be found in S8 Table.

#### Heritability and genetic correlation

We estimated the heritability of our three PWV modes in our study by LD Score regression analysis using LD Hub [38]. To avoid blurring the relationship between PWV and SBP, we used PWV associations only adjusted for age and sex but not SBP. A total of 1,192,954 of our SNPs could be matched to the SNPs available in LD Hub. According to Zheng et al. [38] the minimum criteria to test for genetic correlation are: Heritabilty (H^2^) Z score > 1.5, mean Chi square of the test statistics > 1.02 and intercept estimated from the SNP heritability analysis between 0.9 and 1.1. This held for baPWV but not for bfPWV and cfPWV. We tested for genetic correlation between baPWV and 517 UK Biobank traits and four blood lipids traits (triglycerides, total cholesterol, LDL-cholesterol, HDL-cholesterol) as provided and categorized by LD Hub.

#### Common genetic associations with BP and CAD

To analyse potential patho-mechanistic overlaps between arterial stiffness and CAD, we analysed 169 genome-wide significant CAD-SNPs [1–5] for association with PWV modes in our cohort. We determined the empirical distribution of the number of SNPs associated with at least one of the PWV modes by 10,000 permutations of phenotypes and genotypes. To test for an enrichment, we compared this distribution with the number of actually observed associations with Bonferroni corrected nominal significance (p<0.05/3).

We also wanted to evaluate the genetic relationship between blood pressure and arterial stiffness to gain a better understanding of the underlying common pathomechanisms. For this purpose, we considered 885 SNPs with genome-wide significance for SBP, diastolic blood pressure (DBP) or pulse pressure (PP) described by Evangelou et al. [39] [Supplementary Table 24] and tested them with the same methodology as described above for the CAD SNPs.

Finally we searched for overlaps between BP, CAD and PWV at locus level. For this purpose, we determined the loci for the combined set of CAD and BP SNPs from literature as explained above. A locus containing both, CAD and BP SNPs, is considered overlapping. In addition, a BP / CAD locus was considered overlapping with PWV, when the lead SNP was Bonferroni corrected nominal significant (p<0.05/3) for at least one of the PWV mode.

#### Mendelian randomization

To estimate the causal relationships between blood pressure and arterial stiffness we performed bidirectional Mendelian randomization (MR) analysis considering the genetic associations reported by Evangelou et al. [39] as instruments for blood pressure and the SNPs identified in our GWAS as instruments for PWV. Evangelou et al. used untransformed, medication-adjusted blood pressure traits for their non-genetic model, therefore we decided to recalculate blood-pressure betas in our study for our MR analysis.

To analyse the causal effects of systolic blood pressure on PWV, we excluded the 149 blood pressure SNPs that showed nominal significance for PWV in our data as they represent potential direct effects on PWV which would violate one of the MR pre-conditions. Of the remaining 711 SNPs associated with one or more blood pressure traits, 49 SNPs showed at least nominal significance for SBP in our study cohort and were not in LD with any of our PWV SNPs (all pairwise R^2^ < 0.1). We combined the evidence of these SNPs regarding a causal effect of SBP on our three PWV modes by the inverse‐variance weighted method [40].

To estimate the reverse causal effect of PWV on blood pressure, we used the independent SNPs from our GWAS, which had passed the suggestive significance level for at least one PWV phenotype. None of these SNPs showed a significant effect on blood pressure for our study participants, but one SNP (rs4016640) was in weak LD with a hit reported in Evangelou (rs13082711, R^2^ = 0.11). Therefore we removed this SNP. We then performed MR, applying the inverse‐variance weighted method for each PWV mode separately, using SNPs that showed at least suggestive significance for one PWV mode and at least nominal significance for the PWV mode considered. For a secondary MR, we removed the lead SNP, since there is a weak link between this locus and blood pressure, which could violate the bivariate MR assumption.

**Fig 1:**
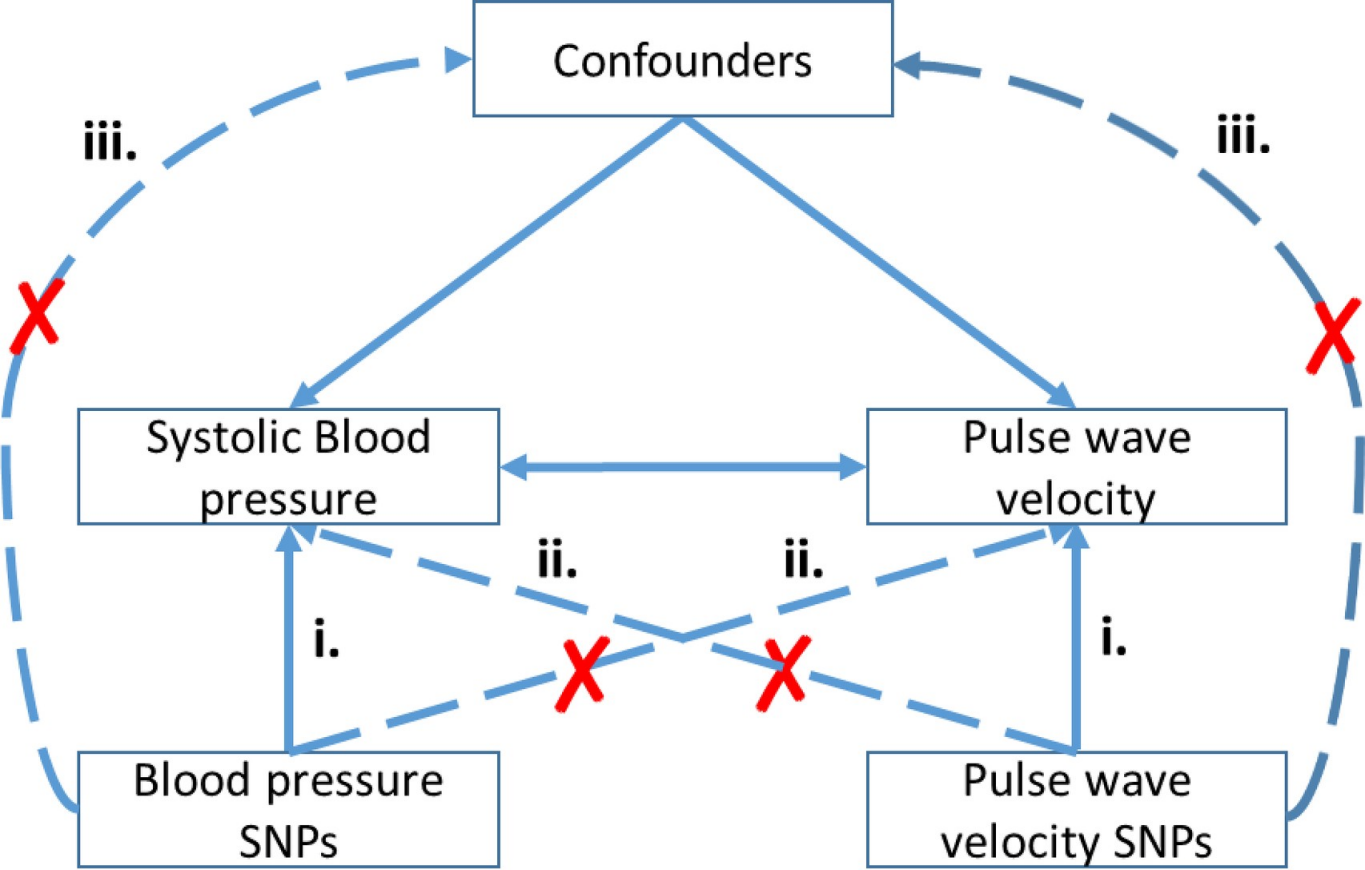
**Bidirectional Mendelian Randomization: To use Mendelian randomization three conditions have to be fulfilled: (i.) There is an association between the SNP and the risk factor, (ii.) the SNP has no association with the outcome and (iii.) the SNP is independent of factors that confound the SNP-outcome relationship.** To analyse the causal relationship of BP on PWV, we use as instruments BP SNPs reported by Evangelou [39], which were also nominal significant in our cohort.

To the best of our knowledge the selected PWV and blood pressure SNPS do not effect confounders for blood pressure and/or PWV [S3c Table in S2 File]. Therefore the preconditions for a bidirectional Mendelian Randomization were fulfilled (Fig 1).

Using Cochrans Q-test to check heterogeneity, we performed a sensitivity analysis of the results to test for pleiotropy effects.

## Results

Basic characteristics of LIFE-Adult and their univariate effects on the three PWV modes are presented in Table 2.

**Table 2:** Upper table: Study characteristics of LIFE-Adult: Analysis is restricted to participants/patients for which genotypes and systolic blood pressures are available.

Study characteristics		Univariate linear regression								
Parameter	LIFE-Adult	baPWV			cfPWV			bfPWV		
		Beta	p-value	Adj. R^2^	Beta	p-value	Adj. R^2^	Beta	p-value	Adj. R^2^
Women / Men (n = 6,758)	3,479 / 3,279	-0.059 (0.004)	<0.001	0.03	0.03 (0.007)	<0.001	0.003	0.09 (0.012)	<0.001	0.02
Age (years)	57.96 (12.62)	0.009 (0.0001)	<0.001	0.43	0.009 (0.0002)	<0.001	0.16	0.01 (0.0004)	<0.001	0.23
Systolic blood pressure (mmHg)	128.94 (16.94)	0.659 (0.01)	<0.001	0.25	0.60 (0.03)	<0.001	0.08	0.67 (0.04)	<0.001	0.06
Diastolic blood pressure (mmHg)	75.42 (9.89)	0.297 (0.02)	<0.001	0.05	0.315 (0.03)	<0.001	0.02	-0.10 (0.05)	<0.001	0.001
**PWV Phenotypes**		**Correlation / p-value**								
baPWV (m/s) (n = 6,734)	10.41 (3.27)				0.37 / 1.86x10^-111^			0.49 / 6.61x10^-214^		
cfPWV (m/s) (n = 6,430)	18.28 (8.10)	0.44 / 2.86x10^-167^						0.51 / 5.0810^−230^		
bfPWV (m/s) (n = 3,643)	18.28 (8.10)	0.52 / 2.93x10^-240^			0.54 / 1.92x10-^257^					

Mean values and standard deviation for parameters of the non-genetic statistical models are presented in column 2. Results of univariate linear regression: Age and SBP explain the largest part of the variance for each of the three phenotypes. For all phenotypes, SBP is stronger associated than DBP. Lower table: (Partial) correlations between PWV phenotypes are shown while controlling for systolic blood pressure (upper triangle) and without (lower triangle). Measuring bfPWV was stopped in June 2013 due to technical reasons, causing lower case numbers compared to baPWV and cfPWV. Remaining difference occur as not all three measures were taken for all patients.

### GWAS results

**Fig 2:**
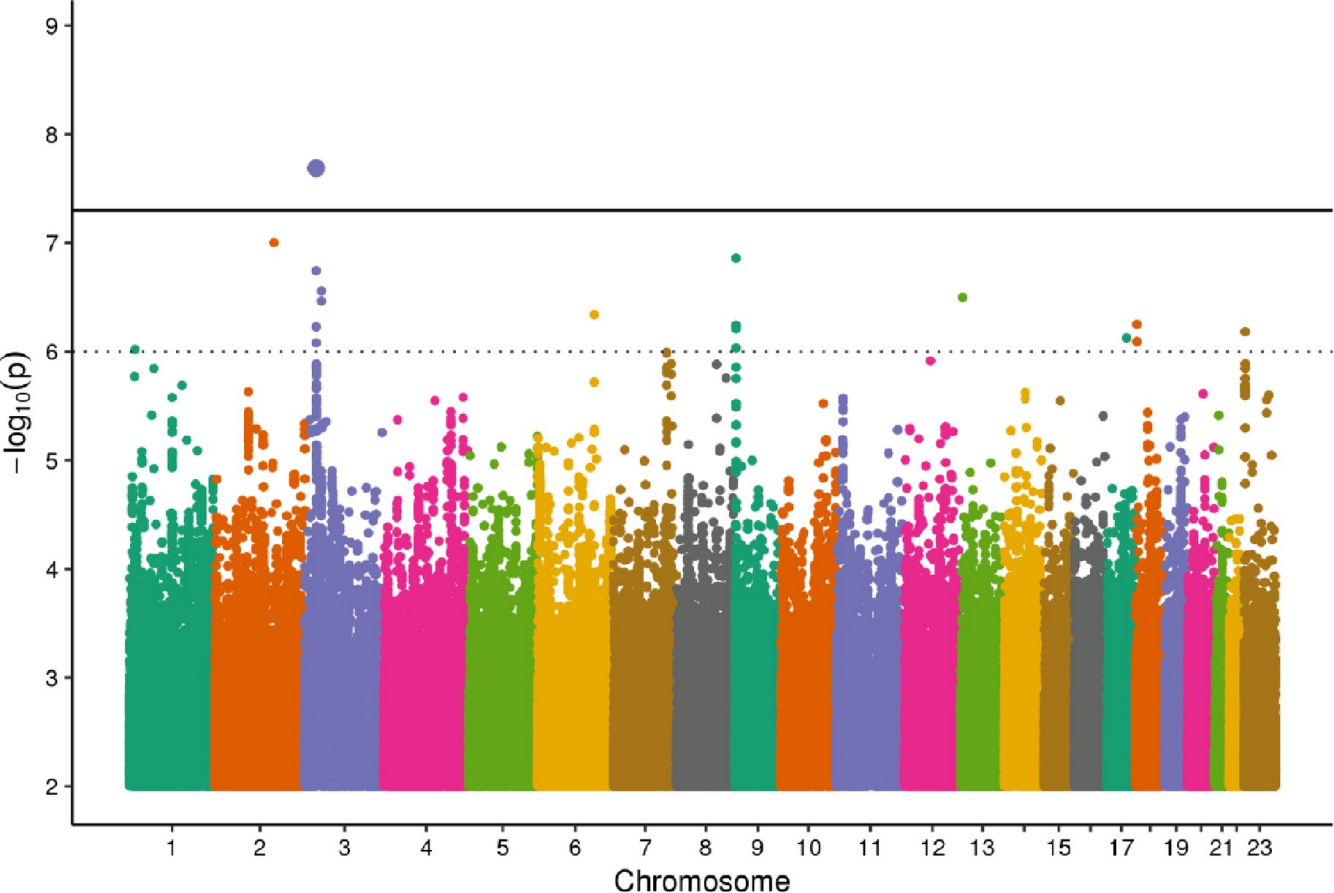
Manhattan plot. Distribution of minimal log10 transformed p-values for all three phenotypes. The bold line marks genome-wide significance (p<5x10^*-8*^). Top SNP rs939834 (Cytoband 3p24.1, phenotype cfPWV) is supported by three SNPs with suggestive significance level (dotted line) in the same locus.

None of three genome-wide analyses showed signs of inflation (λ_cf_ = 1.00, λ_bf_ = 1.01, λ_ba_ = 1.02). We identified thirteen independent SNPs with at least suggestive significance level at 10 different loci. Thereof one locus achieved genome-wide significance [Table 3]. Nine of the eleven suggestive hits reached nominal significance with at least one of the other PWV phenotypes. In all nine cases the beta of the primary and secondary PWV phenotype had the same direction. A Manhattan Plot of all traits is given in Fig 2. Further summary statistics regarding all phenotypes can be found in S4 Table in S2 File. Summary of eQTL look-up and GWAS catalogue look-up can be found in S5 and S6 Tables in S2 File.

**Table 3:** Summary statistics of 10 loci reaching at least suggestive significance for any of the PWV modes.

Cytoband	SNP	Nearest genes (distance)	EA	EAF	Info score	Beta (SE)	p-value / top phenotype	Additional phenotype (p<0.05)
3p24.1	rs1522134	*NEK10* (22 kb); *MINOS1P3* (130 kb); *RNU6-342P* (180 kb)	A	0.19	1.00	-0.026 (0.005)	8.30x10^-7^/ cfPWV	bfPWV
	rs939834*^)^	*NEK10* (75 kb); *MINOS1P3* (180 kb); *RNU6-342P* (230 kb)	T	0.04	1.00	0.058 (0.010)	**2.05x10**^**-8**^**/ cfPWV**	**baPWV**
	rs4016640	*NEK10* (110 kb); *MINOS1P3* (210 kb)	C	0.86	0.97	0.031 (0.006)	5.94x10^-7^/ cfPWV	n/a
2q23.3	rs55895454	*AC104777*.*2* (0 kb); *RND3* (14 kb)	T	0.01	0.63	0.065 (0.012)	9.97x10^-8 /^ baPWV	bfPWV
3p22.3	rs141397708	*STAC* (85 kb); *RFC3P1* (120 kb)	G	0.03	0.98	0.102 (0.020)	2.77x10^-7^/ bfPWV	baPWV
13q12.11	rs35383313	*ZMYM5* (47 kb)	C	0.67	0.82	-0.012 (0.002)	3.18x10^-7^/ baPWV	bfPWV cfPWV
6q22.33	rs74420210	*PTPRK* (0 kb)	T	0.01	0.77	0.190 (0.038)	4.57x10^-7^/ bfPWV	baPWV cfPWV
18p11.31	rs142212026	*LINC00667* (1.7 kb)	A	0.02	0.88	-0.040 (0.008)	5.63x10^-7^/ baPWV	cfPWV
9p24.3	rs77879815	*RP11-443B9*.*1* (2.3 kb); *SMARCA2* (34 kb); *RNU2-25P* (180 kb); *RN7SL592P* (250 kb)	G	0.02	0.96	0.127 (0.033)	5.73x10^-7^/ bfPWV	n/a
	rs77797991	*RP11-443B9*.*1 (1*.*8 kb) SMARCA2 (39 kb) RNU2-25P (190 kb)*	A	0.01	0.85	0.176 (0.057)	1.38x10^-7^/ bfPWV	baPWV
Xp22.32	rs6530472	*Gene desert*	T	0.79	1.00	-0.072 (0.014)	6.58x10^-7^/ bfPWV	baPWV
17q22	17:51577748	*Gene desert*	C	0.01	0.90	0.107 (0.022)	7.51x10^-7^/ cfPWV	bfPWV
1p36.22	rs76651564	*DRAXIN* (0 kb); *MAD2L2* (20 kb); *AGTRAP* (25 kb); *FBXO6* (37 kb); *FBXO44* (48 kb)	A	0.02	0.52	0.192 (0.039)	9.56x10^-7 /^ bfPWV	baPWV

EA: effect allele, EAF: effect allele frequency, info: imputation info score. All summary statistics of all SNPs and phenotypes can be found in S4 Table in *S2 File*.

*^)^ Only rs939834 reached genome wide significance level.

The lead SNP of the genome-wide significant locus was rs939834 (cytoband 3p24.1) near *NEK10* (75 kb), *MINOS1P3* (180 kb), and *RNU6-342-P* (230 kb; all distances with respect to lead SNP). It was best associated with cfPWV (β = 0.058, p = 2.05x10^-8^). There was no significant association with bfPWV (β = 0.009, p = 0.631) and nominal significant one with baPWV (β = 0.014, p = 0.004). Although all three effects have the same direction, effect sizes differed significantly between cfPWV and the other two PWV (comparison of effect sizes for rs939834 using standardized betas and standard errors, p_cf-ba_ = 2.56x10^-3^, p_cf-bf_ = 1.36x10^-5^). The lead SNP was in LD with two cis-eQTL for *EOMES* [33] (highest LD with rs12487751: distance 33kb, LD r^2^ = 0.90, eQTL observed in PBMC and whole blood tissue) and six further eQTLs for *SLC4A7* [33] (highest LD with rs6768039: distance 336kb, LD r^2^ = 0.60, tissue PBMC and whole blood*)*.

Additionally, two independent SNPs, rs4016640 (LD with lead SNP: r^2^ = 8.7x10^-3^) and rs1522134 (LD with lead SNP: r^2^ = 1.2x10^-2^), reached suggested significance at this locus for cfPWV (rs4016640: β = 0.031, p = 5.94x10^-7^; rs1522134: β = -0.026, p = 8.30x10^-7^). Both SNPs are in LD with eQTLs for *NEK10* and *SLC4A7*.

Rs4016640 is in LD with five cis-eQTL for *NEK10* (highest LD with rs1522160: distance 100kb, LD r^2^ = 0.66, tissue heart, left ventricle) and nine cis-eQTL for *SLC4A7* (highest LD with rs67385266: distance 7kb, LD r^2^ = 0.51, tissue blood).

Rs1522134 is in LD with nine cis-eQTL for *NEK10* (highest LD with rs13075192: distance 149kb, LD r^2^ = 0.56, tissue brain, frontal cortex) and nineteen cis-eQTL for *SLC4A7* (highest LD with rs6778395: distance = 50kb, LD r^2^ = 0.94, tissue blood) [33].

**Fig 3:**
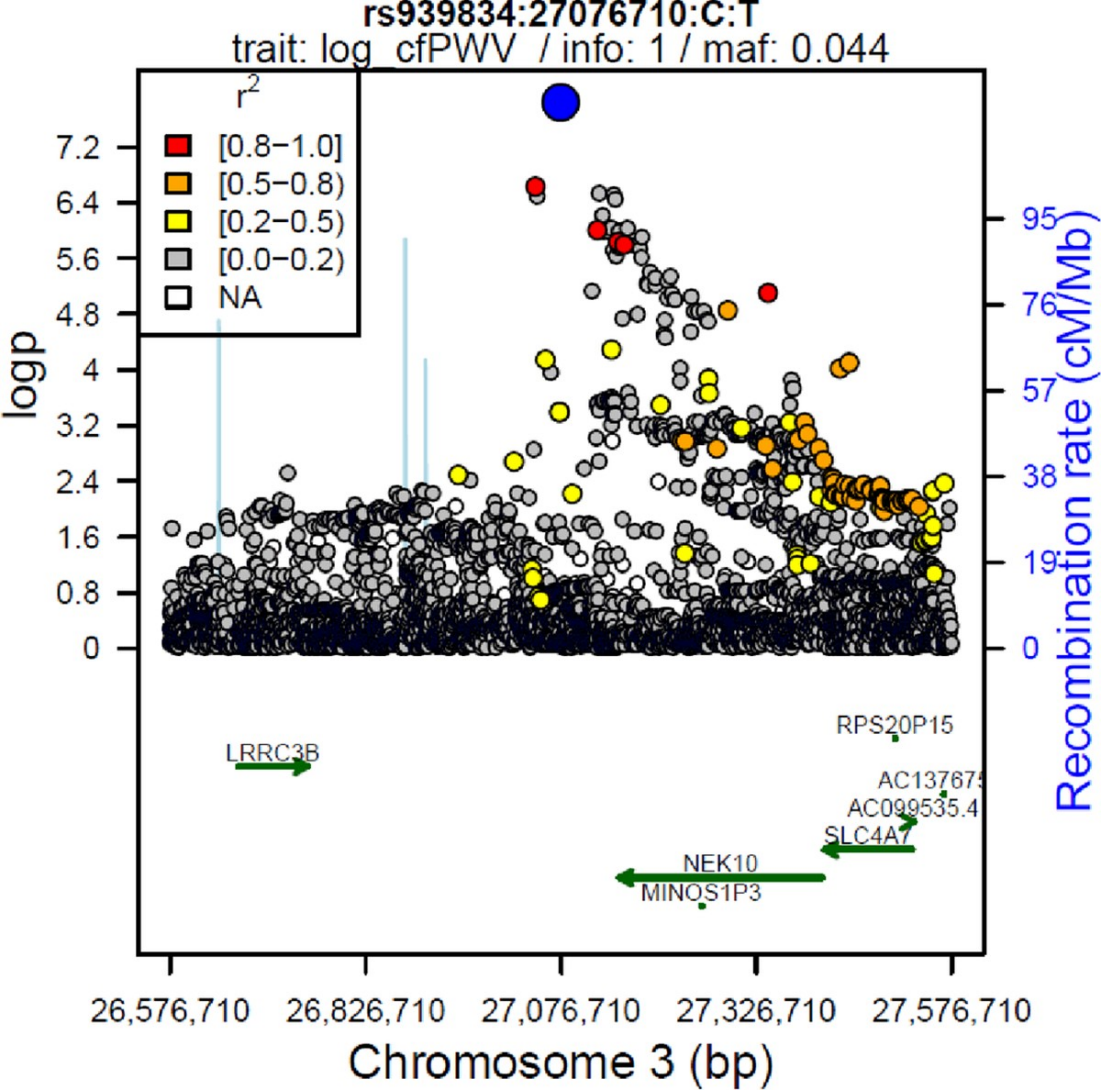
Regional association plot for top SNP rs939834 with cfPWV. The top SNP is coloured blue, the other SNPs according to their LD with the lead SNP). P-values are shown for cfPWV only. The top SNP is strongly supported by additional variants. SLC4A7 as cis-eQTL gene in the same locus is known to effect vascular smooth muscle cells [46].

A regional association plot of this locus is given in Fig 3.

Of the remaining ten suggestive hits, two contain biologically plausible genes: rs55895454 (cytoband 2q23.3) is best associated with baPWV (β = 0.065, p = 9.97x10^-8^) and is close to *RND3* (14 kb), a gene associated with atherosclerosis [41, 42]. There are no eQTLs (LD r^2^ > 0.3) for rs55895454 [33].

The locus on 9p24.3 includes two SNPs. The lead SNP rs77879815 (cytoband 9p24.3) is best associated with bfPWV (β = 0.13, p = 5.73x10^-7^) and close to *SMARCA2* (34kb). It is in LD with one cis-eQTL for *KCNV2* (rs10964107: distance 37kb, LD r^2^ = 0.47, tissue = PBMC and Whole Blood) [33], a protein-coding gene associated with hypertrophy [43].

To confirm the robustness of our non-genetic model, we tested our best-associated SNP rs939834 under further adjustment for antihypertensive and anti-lipid drugs and found no significant change in effect size. We additionally adjusted our top SNPs for log transformed BMI [S7 Table in S2 File]. The results showed no relevant improvements for betas and p-values.

### Replication of known PWV SNPs

Of the 14 candidate SNPs from literature [S8 Table in S2 File], seven showed Bonferroni corrected nominal significance in LIFE-Adult with any of the three PWV modes. Among these seven SNPs, only two were independent in our data. These two independent SNPs (rs17773233 and rs1381289) identified by Mitchell et al. [35] are located at Cytoband 14q32.2 within a distance of 10kb, i.e. they belong to the same genetic locus. SNPs were replicated for baPWV and not for the phenotype of the original study (cfPWV). A comparison of the betas and standard errors for the SNPs between the original GWAS and ours are shown in Table 1. As sensitivity analysis we calculated the effect of each of the SNPs for each PWV mode and with all three published models as well as our own non-genetic model [S9 Table in S2 File], Based on the transformations and non-genetic models described in the original publications no SNP reached nominal significance for their original PWV mode for Park and Tarasov. For Mitchel only rs1461587 (β = -1.95, p = 8.05x10-3) and rs17773233 (β = -2.03, p = 5.79x10-3) reached nominal significance within the male test subjects. No new evidence of replication was detected by this approach.

### Heritability and genetic correlation

We estimated a significant heritability for baPWV (H^2^ = 0.28, 95%-CI [0.16, 0.40], p = 5.6x10^-6^). We also performed a genetic correlation analysis as the minimum requirements were fulfilled for baPWV (H^2^ = 4.54, mean Chi square of the test statistics = 1.04, intercept estimated from the SNP heritability analysis = 1.01). From 517 UK Biobank traits 99 showed nominal significance [S10a Table in S2 File] and thereof three significant genetic correlation after Bonferroni correction (p<0.05/517). “Vascular/heart problems diagnosed by doctor (without high blood pressure, heart attack and angina)” and “fathers age at death” showed a negative genetic correlation (r_g_ = -0.30, p = 2.75x10^-5^ and r_g_ = -0.39, p = 5.79x10^-5^ respectively). “Vascular/heart problems diagnosed by doctor: high blood pressure” showed a positive genetic correlation (r_g_ = 0.27 / p = 6.65x10^-5^). Regarding the four considered blood lipids, triglycerides showed significant positive genetic correlation (r_g_ = 0.27, p = 5x10^-4^) after Bonferroni correction (p<0.05/4).

### Common genetic associations with BP and CAD

Of the 169 SNPs associated with CAD [S11 Table in S2 File], one was not in our data. Of the remaining 168 SNPs, ten SNPs showed Bonferroni corrected nominal significance (p<0.05/3) for at least one of the PWV modes. This represents no significant enrichment compared to an empirical expectation of eight SNPs which showed Bonferroni corrected nominal significance (p<0.05/3) as a result of our permutation analysis (p = 0.18; results were Poisson distributed).

Of the 885 SNPs associated with blood pressure traits [S12 Table in S2 File], 25 were not available in our data. Of the remaining 860 SNPs, 60 SNPs showed Bonferroni corrected nominal significance for at least one PWV phenotype. This represents a significant enrichment compared to an expectation of 41 SNPs (p = 0.002; results were Poisson distributed).

The ten CAD SNPs described above, represented nine overlapping loci between CAD and PWV; the 60 BP SNPs represented the respective number of overlapping BP and PWV loci. Furthermore we identified 110 overlapping loci for CAD and BP, including 9 SNPS that were reported genome wide significant for BP as well as for CAD.

### Mendelian randomization analysis

**Fig 4:**
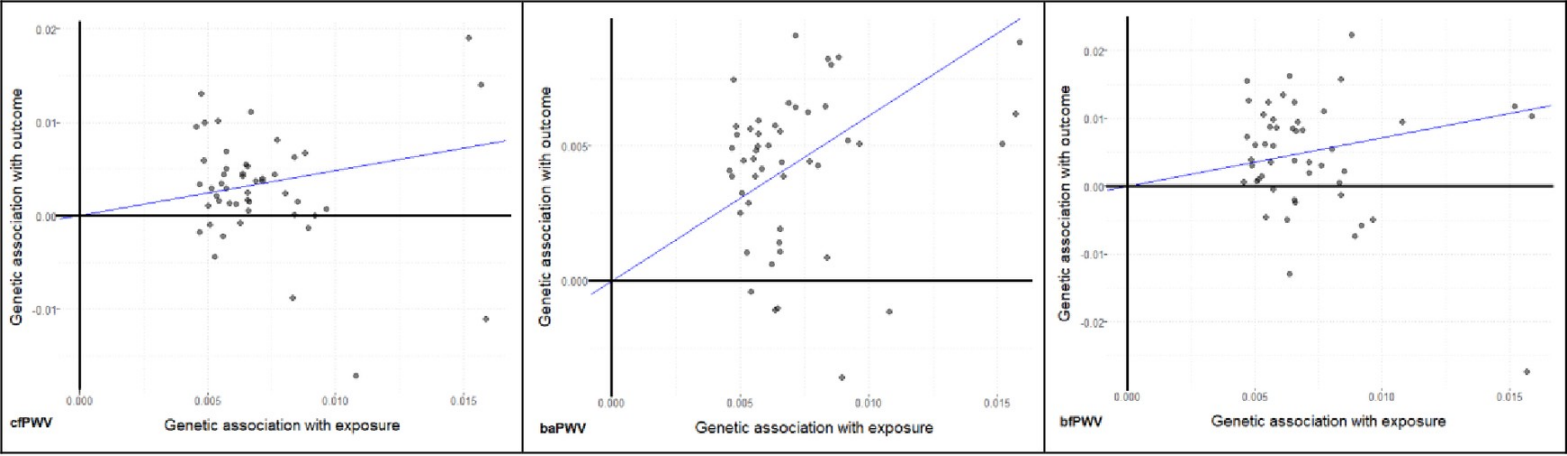
Mendelian Randomization (IVW method): Blood pressure SNPs reported by Evangelou with p-value of less than 0.05 for SPB (and no association with PWV) in our study population were considered as instruments. We report their effect on cfPWV (left), bfPWV (middle) and baPWV (right). Abscissa shows genetic association with BP; ordinate shows genetic association with PWV.

Summarizing the evidence of 49 BP SNPs used as instruments [S3c Table in S2 File], we observed a positive causal effect of systolic blood pressure on all three PWV modes (cfPWV: β = 0.48, p = 1.51x10^-4^; bfPWV: β = 0.72, p = 1.45x10^-3^; baPWV: β = 0.61, p = 6.82x10^-15^) [Table 4]. Genetic association on SNP level is shown in Fig 4. Of note is that for cfPWV and bfPWV there are even single SNPs with nominally significant causal effect.

**Table 4:** Results of Mendelian Randomization analysis. Inverse-variance weighted method was used to combine the evidence of multiple instruments (variants uncorrelated, random-effect model).

PWV	Effect Beta	SE	95% CI LL	UL	p-value
cfPWV	0.481	0.127	0.232	0.730	1.51x10^-04^
bfPWV	0.715	0.225	0.275	1.155	1.45x10^-03^
baPWV	0.612	0.079	0.458	0.766	6.82x10^-15^

The hypothesis that Systolic blood Pressure causally effects PWV was tested. Calculation is based on 49 of the blood pressure SNPs described by Evangelou [39], which showed at least nominal significance for blood pressure in our study population, but no association with PWV.

Vice versa, using 12 PWV SNPs (cfPWV: 4 SNPs, bfPWV: 8 SNPs, baPWV: 5 SNPs) we were not able to show a causal effect of PWV on SBP (cfPWV: β = 0.02, p = 0.76; bfPWV: β = 0.01, p = 0.61; baPWV: β = 0.18, p = 0.86). After removing the lead SNP, which was only significant for cfPWV, the causal effect of cfPWV on SBP was still not significant (cfPWV: β = -0.02, p = 0.81).

Cochrans Q showed no pleiotropy effects for any of the Mendelian randomization analyses performed.

## Discussion

The non-invasively measurable PWV is an established operationalization of arterial stiffness. Performing the first GWAS for three PWV modes in parallel on the same cohort provides unique insights about PWV and the genetic effects on different vascular segments. As the three PWV traits represent different branches of the arterial tree, the significance of the association with the three PWV modes changed for different SNPs. We assume that the association of different SNPs reflect arterial stiffness in different arteries, e.g. SNPs associated with cfPWV act over candidate genes effecting primarily large arteries, whereas baPWV SNPs effect more the peripheral arteries. Accordingly, the three PWV traits are only moderately correlated so that one cannot expect the same genetic background. In addition the variance in the measurements of the PWV modes is different [10], sample sizes for PWV-GWAS are still limited and assessments are note easily comparable [9]. All these effects introduce heterogeneity in the results. While determining arterial stiffness by measuring cfPWV was recommended by AHA scientific statement from 2015 [44], in our study we found higher compliance and higher explained multivariate variance with age, sex and blood pressure for baPWV (adjusted r^2^ = 0.54, p<0.001) compared to cfPWV (adjusted r^2^ = 0.20, p<0.001). Interestingly enough baPWV was also the only PWV mode for which we were able to show heritability.

The SNPs identified in the new genome wide significant locus we discovered at 3p24.1 are all in LD with cis-eQTL SNPs for *SLC4A7* (also known as *NBCn1*). *SLC4A7* encodes a transmembrane protein transporting sodium and bicarbonate ions. According to Boedtkjer et al. [45] *SLC4A7/NBCn1* promotes arterial remodelling in mice. In 2017, Ng et al. [46] published a functional study of variants at the *SLC4A7* locus in cultures of vascular smooth muscle cells (the cellular components of the blood vessel wall) that showed an association between variation at the SLC4A7 locus and blood pressure. The association of this locus with arterial stiffness is therefore biologically plausible.

Biologically interesting are also two loci with SNPs at suggestive significance level. The locus on 2q23.3 around rs55895454 is close to *RND3*, a gene that is assumed to regulate cell actin cytoskeleton dynamics as a negative regulator of cytoskeletal organization, leading to loss of adhesion. A recent study has shown that RND3 can affect signalling in endothelial cells by shifting the balance of RhoA and Rac1 signals [41]. Another study [42] suggests that the integrin–Gα13–RhoA–YAP pathway is a possible target for drug testing against atherosclerosis. Endothelial-cell signalling can affect plaque formation and *RND3* affects, among other things, the RhoA signals in this pathway. The two SNPs in the locus on 9p24.3 are in LD with a cis-eQTL for *KCNV2*, which may affect pulmonary artery smooth muscle cells. Downregulated potassium voltage-gated channels are involved in pulmonary vascular medial hypertrophy associated with pulmonary hypertension [43]. KCNV2 is an inhibitor of functional K^+^ channels and therefore influences the resting membrane potential in vascular smooth muscle cells. Further association studies with larger sample size as well as additional experimental evidence are required to corroborate these associations.

From the six literature loci we analysed, we were able to replicate only the locus “in the 3’-BCL11B gene desert” [35] on chromosome 14 (cytoband 14q32.2). A study published in 2014 [47] showed that *BCL11B* (alias *CTIP2*) might be associated with cardiac hypertrophy (HCM). In 2015 another study showed the correlation between left ventricular hypertrophy and elevated PWV [48], indicating an indirect association between the locus and PWV. None of the other proposed loci was replicated, indicating the heterogeneity of the underlying genetic causes and confirming that the comparability of effects based on different PWV assessments is limited because it also depends on the PWV modes used representing different segments of the arterial tree.

The heritability described for PWV so far is only moderate. While no heritability estimates for baPWV have been published yet, our heritability estimate for baPWV is within the range reported for cfPWV in other literature.

At the current state of research, the relationship between blood pressure and aortic wall stiffening is still not completely understood. While based on the Framingham cohort, higher aortic stiffness was associated with higher risk of incident hypertension, initial blood pressure was not independently associated with progressive aortic stiffening [49]. However, based on the original Framingham Heart Study it was determined that untreated hypertension may accelerate the rate of development of larger artery stiffness [50]. There are other authors that see arterial stiffening as cause for increased blood pressure [51, 52] or expect the functional relationship to be bidirectional [53]. Our permutation analysis confirms a non-random overlap of genetic associations between PWV and blood pressure. Also we showed a positive genetic correlation between high blood pressure and baPWV. Based on the results of our MR we show that blood pressure causally affects PWV. Conversely, we were not able to show a causal effect of PWV on blood pressure.

Limitations: Comparability of our results with those of other studies is still hampered by the different technologies used for PWV assessment. The power of this GWAS differs between our phenotypes due to different sample sizes (between n = 3,462 for bfPWV and n = 6,734 for baPWV). Thus, further GWAS and meta-GWAS are required to identify further robust associations and to corroborate our candidates.

In conclusion, we performed the first GWAS of three PWV modes in parallel and the first for bfPWV at all. We discovered one novel and biologically plausible locus with genome-wide significance and successfully replicated one locus published in literature for another PWV mode. By Mendelian Randomization we established evidence of a causal relationship of BP on PWV but not vice versa. This suggests that arterial stiffness is a consequence but not a cause of increased blood pressure.

## Supporting information

S1 File (DOCX)

S2 File (XLSX)
