# Supplementary material for: Genome-wide association analysis of pulse wave velocity traits provide new insights into the causal relationship between arterial stiffness and blood pressure
Source: PLoS One. 2020 Aug 13;15(8):e0237237. doi: 10.1371/journal.pone.0237237 (PMC7425880; doi:10.1371/journal.pone.0237237)
Supplement: S1 File — (DOCX) [file pone.0237237.s001.docx]

# SUPPLEMENT DATA

## Annotation with expression quantitative trait loci (eQTL) in LIFE-Adult and LIFE-Heart

EQTL-studies designated “LIFE Heart” and “LIFE Adult” in Supplemental Table 3 comprise an update of a previous eQTL-study [1] with larger sample size, summarizing evidence of 4,285 peripheral blood mononuclear cell-samples from LIFE-Heart, and 2,360 whole-blood samples from LIFE-Adult, respectively. Measurement and analysis of the combined data was done as previously described, but now FDR was calculated applying a hierarchical testing procedure based on Benjamini and Bogomolov [2] to avoid excess of false positives on gene level. Furthermore, when jointly analyzing both studies (referred to as "LIFE-Adult&Heart", total sample size 6,645), differences between studies LIFE-Adult and LIFE-Heart were accounted for by including the study identifier as a binary covariate in the regression model.

# SUPPLEMENT FIGURES


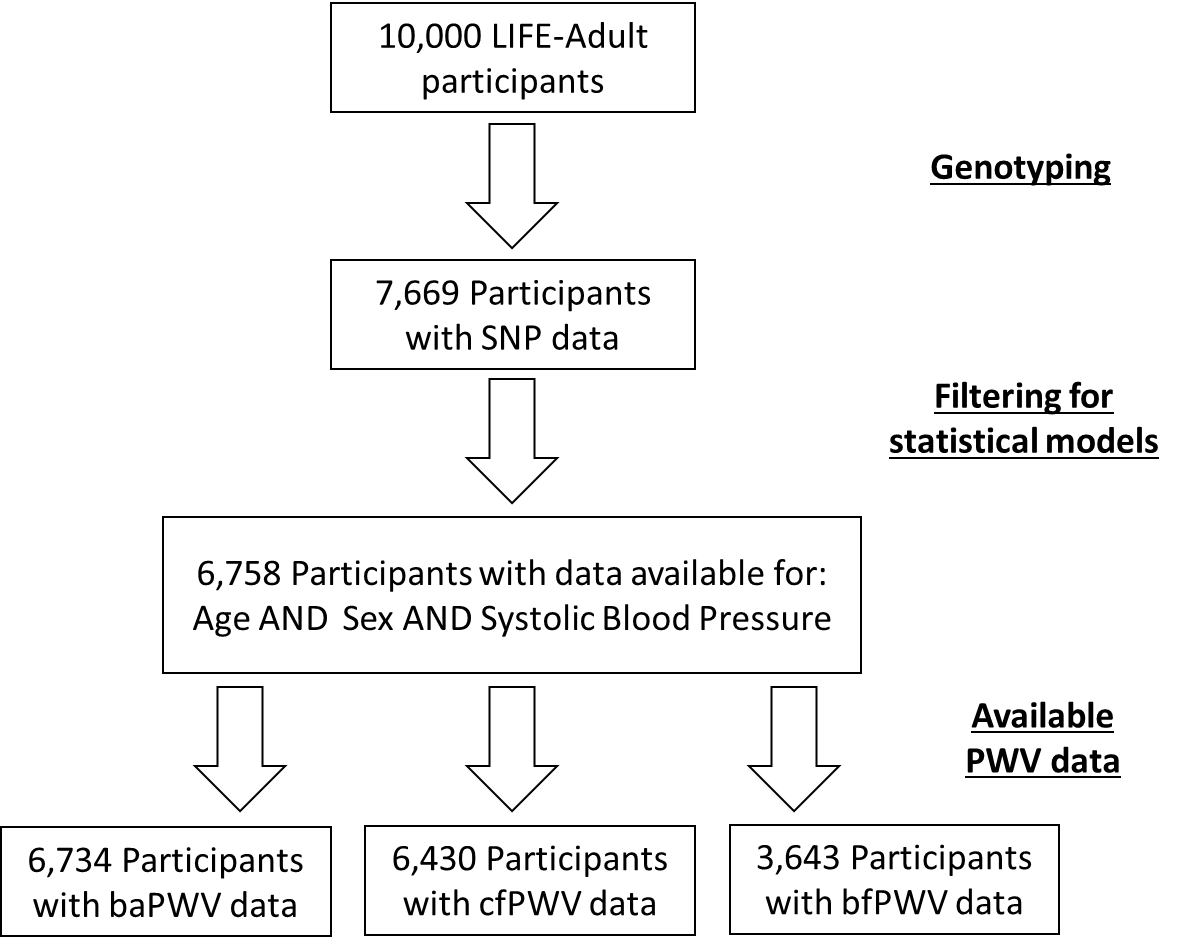


Figure 1: Workflow for selecting input data for GWAS. Only study participants for whom SNP information, PWV data, age, sex and systolic blood pressure was available were selected for GWAS. Number of available data sets for bfPWV was lower than available measurements for baPWV and cfPWV. PWV measurements, genetic data, and required covariates were available for 6,758 participants. Since not all PWV modes were available for each participant, size and composition of the groups for the three phenotypes differed and therefore mean and standard deviation for the co-variables for each phenotype were slightly different.

# REFERENCES

1. Kirsten H, Al-Hasani H, Holdt L, Gross A, Beutner F, Krohn K, et al. Dissecting the genetics of the human transcriptome identifies novel trait-related trans-eQTLs and corroborates the regulatory relevance of non-protein coding loci. Hum Mol Genet. 2015;24:4746–63. doi:10.1093/hmg/ddv194.
2. Benjamini Y, Bogomolov M. Selective inference on multiple families of hypotheses. J. R. Stat. Soc. B. 2014;76:297–318. doi:10.1111/rssb.12028
